# The challenges of managing psychiatric disorders in children with kidney transplant and end-stage renal disease: A case report

**DOI:** 10.1192/j.eurpsy.2021.566

**Published:** 2021-08-13

**Authors:** O. Fodor, A.-M. Ciubotariu, I. Mihailescu

**Affiliations:** 1 Department Of Child And Adolescent Psychiatry, Prof. Dr. Al. Obregia Hospital, Bucharest, Romania; 2 Child And Adolescent Psychiatry, “Alexandru Obregia” Clinical Psychiatry Hospital, Bucharest, Romania

**Keywords:** kidney failure, child and adolescent psychiatry, Mental disorders, Depression

## Abstract

**Introduction:**

Chronic diseases have often been associated with depression or other psychiatric conditions. Despite the fact that renal transplantation offers children a chance at a better life, it could also raise some challenges. Dealing with a severe medical condition such as chronic renal disease and multidrug therapy with potential long term side-effects from the early years of life can affect a child’s emotional and social development.

**Objectives:**

Reporting a case which represents a challenge in treating an adolescent with depression and renal failure.

**Methods:**

Case Report

**Results:**

A 15 year old male with multiple admissions (between the ages of 12 and 15) for recurrent feelings of inadequacy and worthlessness due to his appearance, impaired social skills, hostility towards society, suicide ideation and aggressive behaviour. Moreover, his medical history includes CRS (congenital renal disease), kidney transplant at the age of 2 followed with transplant rejection in 2019. Currently he is under haemodialysis and multiple drug prescriptions associated with his severe medical condition which interferes with the psychotropic treatment.

**Conclusions:**

This case quests what the better choice of intervention is for depression associated with aggressive behavior in a child with kidney failure and with no significant improvement in psychotherapy?

